# Rationale, Design and Methods of “Set the Rules”: A Tailored Peer-to-Peer Health Information Intervention

**DOI:** 10.3390/ijerph15112391

**Published:** 2018-10-29

**Authors:** Jennifer R. Warren, Brandi M. White

**Affiliations:** 1 Community Capacity Builders, Atlantic City, NJ 08401, USA; 2 College of Health Sciences, University of Kentucky, Lexington, KY 40506, USA; brandi.white50@uky.edu

**Keywords:** African Americans, environmental tobacco smoke, health information, community engagement

## Abstract

Ensuring equitable access to health information is one strategy to promote health equity for underserved communities, especially for low-income African Americans (AAs). Childcare centers are one viable site to deliver health information to address this disparity. This paper describes the methods used in a community-based participatory research project with a childcare facility that aimed to reduce environmental tobacco smoke (ETS) exposure among low-income AA children. Through collaboration and multiple data collection methods, partners identified communication strategies to overcome informational barriers. These initial findings indicated a peer-to-peer health information intervention, entitled “Set the Rules”, as the best strategy to increase awareness. The goal of the intervention was to build knowledge in reducing the harms of ETS exposure. Twelve community members were trained as parent leaders for the “Set the Rules” workshops and conducted workshops with parents. Even though there were barriers interfacing with all centers, parents that attended the workshop (*n* = 32) found the peer-to-peer intervention novel and quite helpful and will share the information learned with others. This intervention suggests that a childcare setting is a relevant space to increase access to health information to optimize child health outcomes. More research is necessary to determine if this intervention has salience in other childcare settings and across racial/ethnic groups.

## 1. Introduction

Low-income, minority communities face barriers in access to health information [1,2]. Informational barriers such as these occur when a community’s capacity to provide relevant and useful health information is limited by economic and social factors. These barriers, in turn, affect an individual’s capacity to make effective health decisions about modifiable health lifestyles and behaviors that often exacerbate health problems. As a result, certain groups carry the disproportionate burden of some diseases and illnesses. The solution is not merely to provide information through education. The right opportunities (e.g., situation, place, context) are needed to facilitate the accessibility of information in order to enhance the capacity for behavior change [3]. While communally grounded interventions that involve communities in their development are beginning to address smoking [4,5], less attention has been paid to environmental tobacco smoke (ETS), a problem responsible for an estimated 150,000 deaths each year in the United States (USA) alone [6].

Research consistently demonstrates that ETS is associated with several adverse health effects among children, including asthma [7,8]. The implementation of smoking restrictions by parents has been shown to greatly reduce the negative effects of ETS exposure among young children [9]. Yet, certain groups are still less likely to benefit from the implementation of comprehensive smoking restrictions in which the use of combustible tobacco products is banned. This case study advances the science of community-engaged research through a seven-year participatory research study that aimed to understand, from the parents’ perspective, ETS exposure among young children. This research was conducted in collaboration with a childcare center in Minneapolis, MN, USA. In a previous article, Warren and colleagues provide detailed information on the community-engaged process and main findings [10].

### 1.1. Informational Barriers Related to ETS Exposure Among African Americans

Low-income African-American households are less likely to establish smoking restrictions (SRs) [6]. While there are many barriers influencing SR implementation and ETS exposure, knowledge and healthcare provider communication are highly correlated. Healthcare systems provide opportunities for distributing information on this issue to parents [11]. However, less than half of African-American parents receive advice from their child’s healthcare provider to reduce ETS exposure [10]. Additionally, large-scale studies report that lower-income African Americans tend to have low health literacy in general. This trend is evident in African-American parents scoring lower in knowledge of the harms of ETS exposure compared to White, Hispanic and Asian parents [12]. Gaps in knowledge and inconsistent communication from providers exacerbate smoking-related health outcomes for young, low-income African-American children.

### 1.2. Addressing Informational Barriers for African Americans

Research shows that to address informational barriers, practitioners and/or researchers must first engage relevant African-American communities and capitalize on existing opportunities to disseminate information [13,14,15]. Engaging a trusted community-based organization has the potential to increase awareness of a health issue and to promote information seeking among lower-income African Americans [13].

Community-based, early childcare centers catering to a predominately African-American clientele appear an ideal infrastructure through which to engage African-American parents [10]. Yet, despite the realized benefits of providing health information interventions through early childcare centers, intervention efforts that address children’s ETS exposure in low-income African-American communities are minimal in these settings. Childcare centers provide a context that engages comparable numbers of children and their parents as primary care physicians and pediatricians. Moreover, childcare centers can be a communally salient structure [5] such that staff are reflective of the communities served, including ethnic and neighborhood representativeness, and have awareness of and sometimes participate within the social networks of the parents. Childcare providers understand the values and traditions in which parents and children are embedded, and are often viewed by those communities serviced as a trustworthy place and a beneficial resource for parents (e.g., assisting in subsidy applications, referrals for addressing child health, place to socialize and learn). As a result of these and other factors, interventions by childcare centers have shown to be highly effective in disseminating information that promotes parental behavior change [16,17], which brought about sustainable changes for low-income African-American families [18].

### 1.3. Aims

As a result of these informational barriers, this paper describes the methods used in a participatory study and discusses communication strategies to translate data into a communally relevant health information intervention to reduce ETS exposure among low-income African-American children and promote health equity. What follows is a description of the participatory relationship, and development and testing of the pilot health information intervention.

## 2. Methods/Design

### 2.1. Formative Research

**Figure 1:**
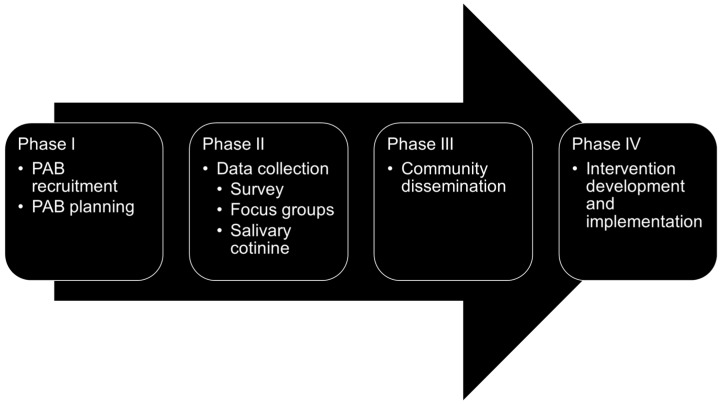
Formative research phases from Parent Advisory Board (PAB) recruitment to intervention development/implementation.

To inform intervention development, four phases occurred during the formative research stage. Findings from these phases have been published elsewhere [10]. Perspectives from parents were used to understand ETS exposure among young children. These methods were as follows (see Figure 1):

- Phase 1: A Parent Advisory Board (PAB) was established. PAB members served as key informants and provided initial insights on smoking and ETS exposure in their community. The PAB also decided on the best data collection methods to gather additional information from the boarder parent community at the childcare center for the next phase.
- Phase 2: Focus groups were conducted with eligible parents at the childcare center to gain further insight on smoking, ETS exposure, gaps in health information, and preferences to receive health information. These findings were used to develop a survey that was administered to the larger parent community. A biochemical measure of children’s salivary cotinine was also taken to assess ETS exposure and to compare with survey reports of the implementation of smoking restriction (see Table 1 for cotinine levels by poverty status). These survey findings have been discussed in greater detail elsewhere [10].
- Phase 3: Survey findings were disseminated to the parent community at a community forum at the childcare center and the larger community at a community event.
- Phase 4: Based on findings from phases 1–2, we developed and implemented a health information intervention, which is discussed in this paper.

### 2.2. Barriers to Accessing ETS Health Information

Findings from the formative research phases identified several factors that were barriers to accessing appropriate and actionable health information related to reducing ETS exposure, especially for young children. These factors are briefly described below.

#### 2.2.1. Individual Factors

Parents were fully aware of the adverse effects of ETS exposure, including thirdhand smoke exposure, exposure through attached housing, and/or knowledge of how to implement smoking restrictions to protect their child.

#### 2.2.2. Interpersonal/Organizational Factors

Survey findings indicated that children’s healthcare providers were inconsistent in providing parents with information about ETS exposure, especially older, nonsmoking parents, or parents who worked fulltime. However, parents were open to their early childcare providers giving them information about ETS exposure.

#### 2.2.3. Community Factors

Many parents who participated in the formative research lived in subsidized housing, which is most often attached housing. This increased the likelihood of children’s exposure to ETS and childhood illness associated with exposure. Parents advocated that health information about ETS should saturate the community and that community members should disseminate this information.

### 2.3. Health Information Intervention

The barriers identified in the formative research phases were used to inform the development of the health information intervention. The findings provided the rationale for continuing the partnership with parents and childcare centers to translate these data into a health information intervention that would disseminate information about the harms of ETS. Community members were included in message and information design, as well as dissemination. Community-campus partners collectively decided to translate scientific findings into a peer-to-peer health information intervention (HII), entitled “Set the Rules”, and train parent leaders to facilitate these workshops for larger parent communities whose children attended Hennepin County Strong Beginnings Childcare Centers in Minneapolis, MN, USA (eight childcare centers).

The use of a peer-to-peer model to disseminate the information was grounded in participants’ desire for someone from the community, in this case parents, to deliver this information. This style of delivery is reflective of a ways of communicating (i.e., information delivery, seeking, sharing) for this African-American community and of held-in-common values [19]. Additionally, the desire to partner with Strong Beginnings is due to the number of centers in Minneapolis as well as the participants’ directive to have information saturate the community. Strong Beginnings is an opportunity structure providing wide diffusion of the information within their centers, which had the potential to infiltrate the social networks of parents attending the session.

The goal of the HII was to build knowledge in reducing the harms of exposure. Five key areas were covered (see Table 2). They were organized in a manual format and contained some or all of the following: handouts, colored posters, visual aids (e.g., bottles with a toxic chemical label), and additional readings to give parent leaders background information. The communally targeted manuals also included information on reducing ETS exposure in attached housing and on reducing and/or removing thirdhand smoke. Sections in the curriculum focused specifically on ETS exposure and smoking restrictions for nonsmokers, older adults, parents who work full time, and noncustodial parents. The curriculum was reviewed for scientific accuracy by a scientific review board, which consisted of a healthcare provider and environmental health specialist.

The translational product was then ready for use in a training session to train parent leaders to deliver the “Set the Rules” curriculum in the identified opportunity structure, childcare settings. Childcare settings allowed for the possibility of greater diffusion, and were identified as reliable and viable sites during the formative research phases. In addition, the location provided an information channel to parents and in some cases access to social networks.

### 2.4. “Set the Rules” Training Sessions for Parent Leaders

#### 2.4.1. Participant Recruitment and Eligibility Criteria

Parent leaders were identified by administrators at the participating childcare centers. These parent leaders would receive training to conduct a “Set the Rules” workshop for the larger parent community at their respective center. Eligibility criteria for parent leaders were that they: (1) have a child between 6 weeks and 5 years of age at a Strong Beginning Center; (2) commit to conducting a workshop at the center their child attends; and (3) self-identify as African American. The recruitment efforts ended with 14 individuals enrolled in the sessions; 10 were parents from Hennepin County Strong Beginnings Childcare Centers. Additional community-based stakeholders requested training. Parent leaders received a $40 stipend and food/beverages for completing the training. On-site childcare was also available.

#### 2.4.2. Procedures

One 2-h training session for parent leaders was held at the community partner’s childcare center. The facilitator conducted a “Set the Rules” workshop with the participants to model techniques and strategies for conducting a workshop. This included use of handouts, posters, flip chart/markers, and props. Participants were divided into smaller groups to model teaching a lesson. Smaller groups also were asked to expand on any information that needed to be tailored for their parent communities. Upon completion, trainees were certified as “Set the Rules” trainers who could then facilitate the workshops at Hennepin County Strong Beginnings Childcare Centers. Both community and academic principal investigators endorsed the certificates. Our goal was to pilot test the “Set the Rules” curriculum to identify changes in knowledge about the harms of ETS and the implementation of smoking restriction.

### 2.5. “Set the Rules” Workshops for Parents

#### 2.5.1. Procedures

Six workshops were conducted over three months. Workshops were held during regular monthly 2-h family night events at the childcare centers. Parent leaders conducted the workshop and administered a pre- and post-assessment of the participants’ knowledge, beliefs and attitudes of the harms of ETS and smoking restrictions, including brief demographic data and an evaluation of their satisfaction with the workshop. The survey used a Likert scale (strongly agree to strongly disagree). The centers provided food/beverages and on-site childcare. The goal was to also recruit a subset of participants attending the training for a 3-month follow up phone call to discern if any behavioral changes were made or if information sharing within familial and social networks occurred. A form was to be disseminated to participants so they could opt in or out of the follow-up study discreetly.

#### 2.5.2. Data Analysis

Descriptive statistics were generated for survey data. Means, medians, standard deviations and ranges will be used to describe continuous variables. Categorical variables were summarized by frequencies and percentages. Additionally, program coordinator field notes were reviewed for salient themes.

#### 2.5.3. Workshop Findings

See Table 3 for demographic characteristics. As for the workshop, the participants highly agreed (range 1.0–2.5) that the content was well organized. This included hearing a description of the objectives for the workshop and those objectives being covered; that the amount of material covered fit the time allotted; and that the content was easily understandable, well organized and helpful. Participants also agreed the workshop improved their overall knowledge of ETS and that they would use the information. The study was unable to collect follow-up information due to challenges with the remote management of a participatory research project.

### 2.6. Ethical Considerations

The ethical review board (Institutional Review Board) at the University of Minnesota—Twin Cities approved the research protocol (#0802E26361).

## 3. Discussion

This article sought to advance the science of community-engaged research by describing the methods of a participatory study that aimed to reduce informational barriers related to ETS exposure for young, African-American children. Parents and childcare centers provided context and an in-depth understanding of the barriers to accessing ETS health information. Subsequently, these findings facilitated the development and dissemination of a community-sanctioned HII, titled “Set the Rules”. This approach fundamentally drew upon parents’ perspectives as part of the process to determine communication strategies and to translate data into relevant health information. We do not yet know if “Set the Rules” works, so we are unable to conclude anything about the efficacy of this intervention.

To our knowledge, this is the first article to highlight a participatory partnership with childcare providers to: (1) identify determinants of ETS exposure; (2) determine parental knowledge gaps and informational barriers; and (3) implement a theory-based intervention to address barriers surrounding the harms of ETS among young children. Methodologic triangulation established rigor by affording a matrix of factors to ascertain points of saturation—the repetition of certain data points across parents and methods that facilitated the development and implementation of a targeted HII.

“Set the Rules” integrated communication strategies that capitalized on existing social ties and networks within the community, and of particular interest, those between parents and the childcare centers servicing their communities and families. There was a trustworthy relationship between childcare administrators and parents. As a context, these centers afforded a safe space to discuss ETS exposure, which could make parents who smoke feel uncomfortable. In the process of conducting this research, childcare centers did emerge as a viable mode to deliver the information. Centers provided access to parents, and administrators were diligent to see that enrollments for different data collection phases were successful. This was also the case with the biomedical measure of cotinine from children. Research has shown that African Americans tend to mistrust this type of science. Yet, the childcare centers were successful in parent recruitment.

The peer-to-peer approach of the HII capitalized on existing interpersonal relationships and social influence [1]. While the longitudinal impact of the HII on behavior could not be ascertained in this pilot study, the information communicated through the “Set the Rules” format increased parental knowledge of the harms of ETS. As a health literacy tool, “Set the Rules” may have relevance as a community-usable product. Further research is needed to evaluate the relationship of the curriculum and delivery method to behavioral outcomes across a larger population of parents that hold similar demographic characteristics to the parents in this study.

### 3.1. Barriers to the Implementation of the “Set the Rules” Workshops

There were salient points noted in the field notes during the implementation of “Set the Rules”. First, it was very difficult to coordinate with participating childcare centers. The community partner had to assist in facilitating contact between the program coordinator and childcare administrators. Second, not all of the childcare sites wanted to use a family night to conduct a “Set the Rules” workshop. A few childcare administrators set up the event on site with a few chosen participants. Some sites adapted the teaching by having more than one facilitator deliver the curriculum, in addition to a request to have the information intervention target Native American Indians. Other childcare centers shared the “Set the Rules” across a variety of programs aligned with the sites (e.g., pregnant mothers, parenting groups) and in other informal ways with family and friends. Third, the timeframe for conducting the workshops conflicted with childcare administrators being busy and balancing staff responsibilities that occur during the fall. Fourth, childcare centers had some difficulty recruiting families for training due to the perceived sensitive topic of ETS. Fifth, childcare administrators felt the curriculum had the potential to motivate and assist parents in advocating for safe and healthy public housing policy.

### 3.2. Study Limitations

This study had several limitations. Childcare administrators were overwhelmed with competing priorities, including administrative responsibilities related to federal subsidies to pay for childcare. Administrators were also advocates, working extremely hard to provide stable care for children. As a result, when working with this community, some parents were lost to the system. A rolling recruitment for the Parent Advisory Board enabled access to parents throughout the participatory research process. 

Because the study targeted specific childcare centers, sample sizes were small and the findings may not be generalized across all low-income, African-American parents, families and communities addressing ETS exposure among young children. Focus groups were also small and not stratified by age group. Future research in this area could stratify focus groups by age group in order to identify different age- or generation-specific techniques to address community-level health issues. In addition, pre- and post-tests were also not collected to assess quantitative changes in knowledge for participants who attended “Set the Rules” workshops facilitated by parent leaders. Data collection was also challenging because of competing priorities, misunderstandings about the research process, and inadequate staffing.

## 4. Conclusions

Despite the limitations, a communally grounded, participatory process and ecological view of a health phenomenon informed by rigorous methodological praxis is the first step to translating data into local, relevant and possibly useful information interventions to reduce information barriers that facilitate tobacco-related health disparities for low-income African Americans. The described case study can be used as a template for other collaborations with childcare centers conducting research on child health issues. Moreover, engaging community partners throughout the research process can build community interest to be involved in and to conduct research, as well as to increase the capacity for sustainability.

## Figures and Tables

**Table 1:** Cotinine level based on poverty status (*n* = 43).

Cotinine Level	Under Poverty Level	Above Poverty Level
Nondetectable	6%	44%
Low	11%	12%
Intermediate	28%	16%
High	56%	28%

**Table 2:** “Set the Rules” content.

Lesson	Key Content
1	What is environmental tobacco smoke?
2	How are we exposed to environmental tobacco smoke?
3	What toxic chemicals are in environmental tobacco smoke?
4	What are the harm effects caused by environmental tobacco smoke?
5	How do I set smoking restrictions?

**Table 3:** Demographic characteristics of “Set the Rules” workshop participants.

Characteristic	Percent/Mean
Female, %	100%
Age, Mean	35 (range: 21–56)
Number of children 5 years and younger, Mean	1 (range: 1–4)
Employment status: not currently working, %	42%
Smoking restrictions, %	
Complete home smoking ban	78%
Complete car smoking ban	70%
